# Forecasting Caseload of Critically Ill Patients Who Are Alert and Without Delirium for at Least Two Consecutive Days for the Assessment of Their Psychological Distress

**DOI:** 10.7759/cureus.39859

**Published:** 2023-06-02

**Authors:** Rachel A Hadler, Franklin Dexter

**Affiliations:** 1 Anesthesiology, Emory University, Atlanta, USA; 2 Anesthesia, University of Iowa, Iowa City, USA

**Keywords:** forecasting caseload, workforce planning, operations research, palliative care delivery, intensive care unit stay

## Abstract

Introduction: One-quarter of alert, non-delirious patients in critical care units report significant psychological distress. Treatment of this distress depends upon identifying these high-risk patients. Our aim was to characterize how many critical care patients remain alert and without delirium for at least two consecutive days and could thus predictably undergo evaluation for distress.

Methods: This retrospective cohort study used data from a large teaching hospital in the United States of America, from October 2014 to March 2022. Patients were included if they were admitted to one of three intensive care units, and for >48 hours all delirium and sedation screenings were negative (Riker sedation-agitation scale four, calm and cooperative, and no delirium based on all Confusion Assessment Method for the Intensive Care Unit scores negative and all Delirium Observation Screening Scale less than three). Means and standard deviations of means for counts and percentages are reported among the most recent six quarters. Means and standard deviations of means for lengths of stay were calculated among all N=30 quarters. The Clopper-Pearson method was used to calculate the lower 99% confidence limit for the percentages of patients who would have had at most one assessment of dignity-related distress before intensive care unit discharge or change in mental status.

Results: An average of 3.6 (standard deviation 0.2) new patients met the criteria daily. The percentages of all critical care patients (20%, standard deviation 2%) and hours (18%, standard deviation 2%) meeting criteria decreased slightly over the 7.5 years. Patients spent a mean of 3.8 (standard deviation 0.1) days awake in critical care before their condition or site changed. In the context of assessing distress and potentially treating it before the date of change of condition (e.g., transfer), 66% (6818/10314) of patients would have zero or one assessment, lower 99% confidence limit of 65%.

Conclusions: Approximately one-fifth of critically ill patients are alert and without delirium and thus could be evaluated for distress during their intensive care unit stay, mostly during a single visit. These estimates can be used to guide workforce planning.

## Introduction

Although psychological distress is prevalent among patients experiencing and survivors of critical illness, strategies are limited for screening and treating distress in intensive care unit patients [1,2]. Recent studies have emphasized the potential value of providing psychological and/or psychiatric support to high-risk individuals [3-5]. At the studied hospital, such support often is provided by consultative nursing services: psychiatric nursing and supportive care nursing.

The Patient Dignity Inventory [6] has been used to evaluate the potential to use patient and intensive care unit characteristics to predict intensive care unit patients with severe distress [1]. The Patient Dignity Inventory assesses psychological, physical, and social stressors in patients nearing the end of life [7]. The tool also is valid and psychometrically reliable when used among critically ill patients who are alert and without delirium [8]. The scoring is one “not a problem”, two “slight problem”, three “a problem”, four “a major problem”, and five “an overwhelming problem” [1,6,8].

Psychological distress is common among intensive care unit patients. Among 155 intensive care unit patients completing 3857 items, there were 23% (906) items with scores of three, four, or five [8]. There were 34% of the patients (52/155) with severe dignity-related distress [1]. However, none of the covariates was reliably associated with Patient Dignity Inventory scores [1]. Multiple combinations of informative predictive models and potential covariates all had incorrect classification rates of at least 34% of patients (52/155) because all methods maximized accuracy when predicting that no patient had severe distress [1]. The investigators concluded that all critically ill patients alert and without delirium for at least two days should be evaluated for distress [1], using the Patient Dignity Inventory or another validated instrument such as the Hospital Anxiety and Depression Scale. Additional predictive modeling showed that care models for the assessment and treatment of these intensive care unit patients with dignity-related distress should not rely solely on the intensive care unit team but instead should be taken from the perspective of the entire hospitalization [2].

The goal of our retrospective cohort study was to estimate the resulting increase in mental health practitioner caseload:

1. How many new patients alert and without delirium for at least two days can be identified daily, expressed as a rate for the study hospital and as a proportion of all intensive care unit patients for generalizability to other hospitals?

2. How many consecutive days are these patients typically alert, without delirium, and in the intensive care unit?

We considered critical care patients who were alert and without delirium for at least two consecutive 24-hour periods, 12-noon to 12‑noon to 12‑noon (e.g., Sunday 15 January 2022 at 12:00 through Tuesday 17 January 2022 at 12:00). The interval may be important when considering treatment options for dignity-related or psychological distress, as interventions such as psychiatric nursing or palliative care consultation typically involve multiple assessments [9,10].

## Materials and methods

The University of Iowa Institutional Review Board approved this retrospective cohort study, 201911151, “Predictors of Anesthesia Department Patient Outcomes”, without requiring patient consent.

The studied hospital has three adult intensive care units: surgical, cardiovascular, and medical. The study’s functional starting date was the fourth quarter of 2014 when the patients were consistently meeting all inclusion criteria. The study end date was the first quarter of 2022, the last full quarter before data analysis. The studied total patient care hours, and the studied counts of patients, both referred to all patients in one of the three intensive care units. The inclusion criteria for the study patients were adults (defined as patient age >17 years on the date of hospital admission), present in an intensive care unit at 12 noon one day and continually so for the next 48 hours, who during that 48 hours or longer interval had every Riker sedation-agitation scale four, “calm and cooperative”, and during those two days had all Confusion Assessment Method for the Intensive Care Unit scores negative (i.e., no delirium) and Delirium Observation Screening Scale less than three (i.e., no delirium) [2]. Alertness was assessed several times daily, and delirium assessments were made at least daily. If the Riker scale was missing, or if both Confusion Assessment Method for the Intensive Care Unit and the Delirium Observation Screening Scale scales were missing, the criteria were considered as not satisfied. Intubation was not considered an exclusion criterion if the documentation was consistent with the patient’s ability to take part fully in the delirium assessment, and the patient was not delirious. The criteria excluded the patients admitted to an intensive care unit for brief (e.g., overnight) monitoring. Only the first admission meeting criteria were included for each patient.

Spearman rank correlation coefficients were calculated among the N=30 quarters studied. To test whether the fraction of patients awake increased over time (e.g., due to initiatives to reduce sedation and maintain wakefulness for delirium prevention [11]), correlation coefficients were compared with zero using one-sided tests. P <0.01 was treated as significant.

Means and standard deviations of means for counts and percentages were calculated among the first six quarters studied and among the last six quarters. For these statistics, the primary results were the Spearman rank correlation coefficients, as tested inferentially. The counts and percentages were provided for context, not for inference. Means and standard deviations of means for lengths of stay were calculated among all N=30 quarters. Again, these are standard deviations among quarters, not among patients, because the goal of these analyses was to judge workload.

The conservative, exact Clopper-Pearson method was used to calculate the lower 99% confidence limit for the percentages of patients who would have had at most one assessment of dignity-related distress before intensive care unit discharge or change in mental status.

## Results

From October 2014 to March 2022, the hospital increased intensive care unit beds (Spearman correlation coefficient 0.56), from 74 (standard deviation 3) to 76 (0) beds. Patient care hours (i.e., bed occupancy) increased during that period (Spearman correlation 0.63), but admissions did not (Spearman correlation 0.13).

During the last six quarters, there was an average of 3.6 (standard deviation 0.2) newly awake patients per calendar day. There were at least two newly awake patients on 84% of days (458/547) and at least three on 66% of days (363/547). Those percentages mean the study hospital would achieve 80% statistical power with a survey (e.g., using its electronic health record data) of N=20 days (or N=100 days) to make one-sided tests that most days (>50%) have at least two new patients (or three new patients), respectively.

The percentages of intensive care unit hours among patients awake for at least three successive 12-noon (i.e., 48 hours) decreased slightly (Spearman correlation ‑0.58, P=0.0004), from 22% (standard deviation 1%) to 18% (2%). The percentages of intensive care unit admissions among patients who were alert and without delirium for at least three successive 12-noon during their admission decreased slightly over the 30 quarters (Spearman correlation ‑0.46, P=0.005), from 22% (standard deviation 2%) to 20% (2%). This wakefulness rate of at least 20% answers our first question from the perspective of other hospitals.

Among the 10,314 awake patients, there was no significant change over time in the duration of alert, without delirium, and in an intensive care unit (Spearman correlation 0.04). The period when alert and without delirium started a median of 2.3 days into hospitalization (25th percentile 1.3, 75th percentile 5.0). The average time alert and without delirium before transfer to the floor, development of delirium, or another change in clinical status (e.g., respiratory decompensation requiring intubation and sedation) was 3.8 days (standard deviation 0.1), with a median of 3.08 days (25th percentile 2.2 and 75th percentile 4.4). We applied these answers to our second question in the context of daily assessments of distress and intervention before the date of change of condition (e.g., transfer). Any patient alert and without delirium in the intensive care unit would be exposed to an average of 1.7 (standard deviation 0.1) assessments, with 63% (6488/10314) of patients having zero or one assessment, lower 99% confidence limit 62%. If assessments were made every 48 hours during a patient’s intensive care unit admission, there would be an average of 1.0 (standard deviation 0.1) assessments per patient, with 77% (7958/10314) of patients having zero or one assessment. Most patients remained hospitalized for many more days (Table 1).

**Table 1:** Characteristics of the 10,314 critical care patients alert and without delirium for at least two consecutive days (three consecutive 12-noon)

Characteristic	% (N)	Median (25^th^, 75^th^ percentiles)
Female	41% (4195)	
White	89% (9178)	
African American/Black	6% (581)	
Hispanic/Latino of any race	3% (272)	
Asian	1% (120)	
Other, each of the six responses <1%	3% (283)	
Surgery anytime during admission before time awake in intensive care	40% (4135)	
Surgery within a week beforehand	37% (3827)	
Days admitted to hospital before the 12-noon starting the first period awake		2.3 (1.2, 5.0)
Days hospitalized		12.5 (7.9, 20.6)
Days in intensive care unit during visit when awake		4.9 (3.5, 7.1)
Surgical intensive care unit	39% (4056)	
Cardiac intensive care unit	32% (3333)	
Medical intensive care unit	28% (2925)	
Discharged to home with self-care	36% (3761)	
Discharged to skilled care facility	17% (1716)	
Discharged to inpatient rehabilitation facility	14% (1423)	
Discharged to home with home health care	9% (934)	
Deceased, not discharged alive	9% (899)	
Discharged to long-term acute care hospital	6% (615)	
Discharged to hospice, medical facility	2% (229)	
Discharged to hospice, home	1% (141)	
Discharged to skilled swing bed unit	1% (107)	
Discharge to a location different than the preceding nine rows	5% (489)	
Narcotic during the first 24 hours of the studied two or more consecutive days	29% (3034)	
Oxycodone	18% (1814)	
Fentanyl	7% (747)	
Hydromorphone	4% (453)	
Morphine	3% (279)	
Hemodynamic infusion during the first 24 hours of the studied two or more consecutive days	27% (2741)	
Norepinephrine	14% (1407)	
Nicardipine	9% (879)	
Vasopressin	5% (510)	
Epinephrine	5% (498)	
Milrinone	2% (244)	
Ventilatory support during the first 24 hours of the studied two or more consecutive days	26% (2725)	
Mechanical ventilation	13% (1367)	
Optiflow^TM^ nasal high-flow therapy	10% (1064)	
BiPap, bilevel positive airway pressure	3% (294)	
Cardiovascular support during the first 24 hours of the studied two or more consecutive days	3% (325)	
Ventricular assist device	2% (162)	
Intra-aortic balloon pump	1% (122)	
Extracorporeal membrane oxygenation	0.4% (43)	
SARS-CoV-2 positive	1% (92)	

## Discussion

The goal of our retrospective cohort study was to evaluate how to forecast the caseload of critically ill patients who are alert and without delirium for at least two consecutive days for the assessment of their psychological distress. Our findings on patient days spent alert, without delirium, and in an intensive care unit, imply that most patients meeting inclusion criteria would be assessed one time before transfer. This result is practical for forecasting new psychological/mental health worker caseload because the number of visits probably will be close to proportional to the number of new patients meeting the criteria we studied. Furthermore, if the intervention(s) were made to treat distress, the interventions would likely be short in duration (e.g., starting with the administration of the Patient Dignity Inventory or other instruments in a reflective manner) [12].

Earlier survey studies examined distress in intensive care unit patients [1,8], not long-term outcomes. At 0.5 to 1.5 years after intensive care for trauma, 30% of patients (71/238) have a clear recollection of the whole intensive care unit period [13]. One year after intensive care unit discharge, severe post-traumatic stress disorder was diagnosed among 27% (48/180) of patients, anxiety among 18% (35/192), and depression among 12% (22/192) [14]. If there were an association between dignity-related distress and post-intensive care unit syndrome, and if treatment of the dignity-related distress often successfully reduced the distress and that had long-term benefits, then the treatment would have better cost utility. On the other hand, severe psychological distress (i.e., prefer death to the current condition) should be treated regardless, if the cost of the therapy is low and without adverse effects.

Our study reflected practice patterns at a single hospital. Given the large population (>10,000 patients) and lengthy period studied (eight years), we doubt this was an important limitation in terms of estimating average days awake among patients. While the incidence of daily cases will vary among hospitals, the point in terms of estimating associated caseload (e.g., for a consultative mental health support service) is that the proportion of patients who are alert and without delirium was substantive (approximately one-fifth). There were only 1% of intensive care unit patients alert and without delirium for at least two consecutive days (including three 12-noon) and with coronavirus disease 19 (Table 1). When not sedated and without delirium, these patients generally were discharged to coronavirus disease 19 pulmonary ward(s) and thus were not included in our study data.

Finally, for clinicians choosing to use the Patient Dignity Inventory [6], typical times for administration in a reflective manner are approximately 30 minutes [1,8,12]. The hospital studied would engage psychiatric nurses and other specialists with psychiatric, psychological, or palliative care training to optimize the efficacy and timeliness of such interventions. Although some hospitals may choose to train intensive care unit team members to provide such primary palliative care services, we showed previously using machine learning modeling that individual patients cannot be predicted accurately to remain in the intensive care unit and therefore planning should be done from the perspective of the entire hospitalization [2].

## Conclusions

A substantive proportion of intensive care unit patients are alert and without delirium, approximately 20%. Daily assessment and treatment for these patients’ psychological distress would be brief because two-thirds of these patients would have at most one such assessment. These two managerial epidemiology results can be used for judging the increase in palliative care (psychological) caseload when an assessment program is implemented. Future studies can quantify the burden of identifiable dignity-related distress among these patients.
